# Colorectal Carcinoma in Childhood

**DOI:** 10.1097/PG9.0000000000000039

**Published:** 2020-12-17

**Authors:** Esra Polat, Nevzat Aykut Bayrak, Engin Tutar, Cigdem Celikel, Gulnur Tokuc, Deniz Ertem

**Affiliations:** From the * Division of Pediatric Gastroenterology, Hepatology, and Nutrition; † Division of Pediatric Pathology,; ‡ Department of Oncology, Marmara University School of Medicine, Istanbul, Turkey.

Colorectal cancer (CRC) is very rare those younger than 20 years of age with an annual incidence of 1 case per million individuals (1). While a majority of the published CRC cases were older adolescents, reports including pediatric CRC series are limited in number (2,3). Hence, CRC is overlooked in the differential diagnosis of unexplained abdominal pain, weight loss, and anemia in children (4). At presentation, children with CRC usually are at advanced-stages of the disease and have a higher frequency of unfavorable histopathology, consequently poor outcome compared with adults (3).

## CASE REPORT

**FIGURE 1.**
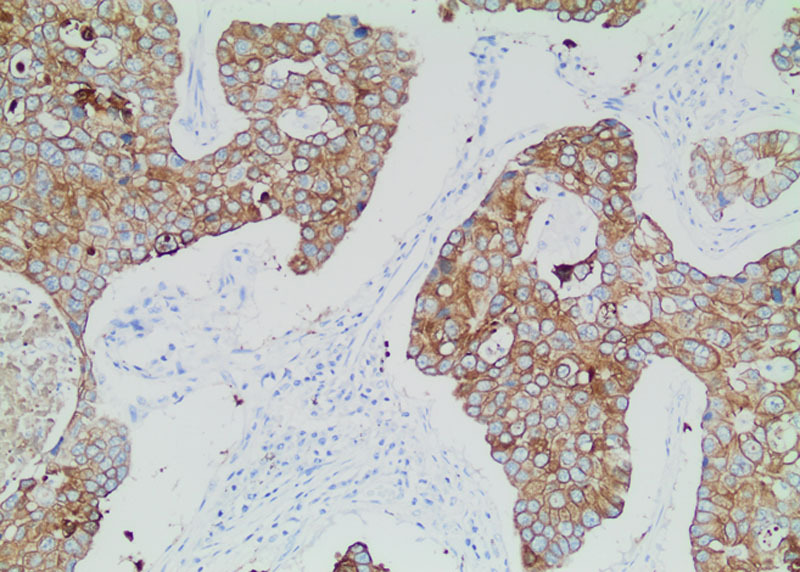
Poorly differentiated adenocarcinoma. Isolated and small group of neoplastic cells within mucin pools, as well as neoplastic cells that partially lining the mucin pools (MOC-31; X20).

**FIGURE 2.**
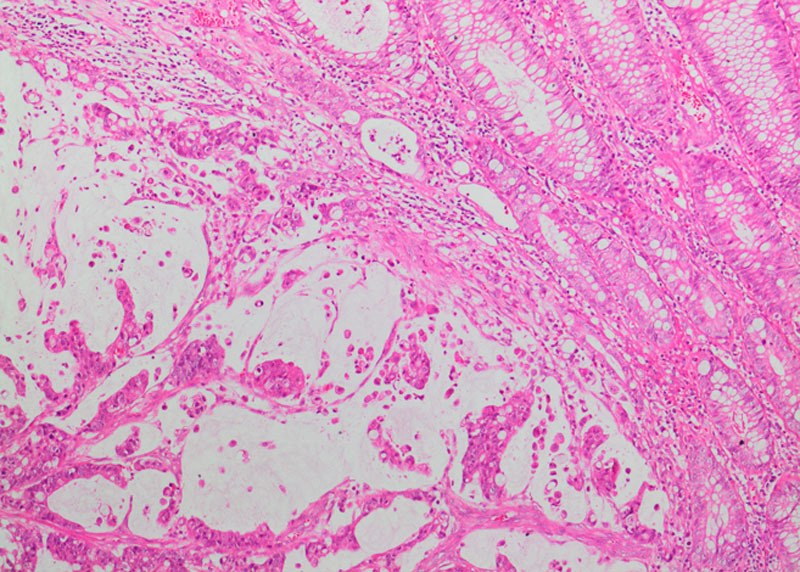
Mucinous adenocarcinoma. Malignant loosely cohesive epithelial cells as solid and gland-like structures surrounded by desmoplastic stroma (H&E; X20).

Five patients who were diagnosed with CRC between 2013 and 2018 in a single pediatric gastroenterology center were retrospectively reviewed with respect to demographic data, presenting symptoms, endoscopic, histopathological, radiologic characteristics: medical and surgical interventions and outcomes (Table 1). All the patients were male except 1 patient. The youngest one was diagnosed at age of 12. Bloody and mucous stool was the most common symptom and, 3 out of 5 of them had prominent weight loss as well. Only 1 patient (patient no. 1) expressed abdominal pain. The duration of symptoms varied between 2 and 6 months except for the patient (patient no. 2) with intermittent bloody defecation and significant weight loss for 12 months. The family history was remarkable in none of the patients, regarding the history of colorectal malignancy/polyposis syndromes or the presence of consanguinity. Since the presenting symptom was blood in stool in all, a colonoscopy was carried out at first. Colonoscopy revealed a rectal solid mass in 3 patients, and a mass in descending colon in the remaining 2 patients. The solid mass did not cause a full obstruction in any patient. A repeat colonoscopy in patient 4 after left hemicolectomy, revealed adenomatous polyps with high-grade dysplasia in transverse colon and cecum. Abdominal magnetic resonance imaging (MRI) was carried out in all patients, and 1 patient (patient no. 1) had a liver metastasis at diagnosis. Histopathology of the rectal mass in this patient (patient no. 1) was compatible with poorly differentiated adenocarcinoma (Fig. 1). Histopathology of the tumors confirmed a mucinous adenocarcinoma in the remaining 4 patients (Fig. 2). Surgical resection of the colonic solid mass was performed in 4 patients, the family of 1 patient (patient no. 2) refused treatment. After the resection of the tumor, all patients received chemotherapy, and 1 patient (patient no. 4) also received radiotherapy.

**TABLE 1.** Demographic Data and Characteristics of the Patients

Patient Number	Age (yr)	Sex	Symptom	Duration of Symptoms (mo)	Colonoscopy Findings	Tumor Location	Metastasis	Histopathology	Treatment	Outcome
1	12	M	Bloody stool, abdominal pain	3	Rectal solid mass	RS	Liver	PDA	STx+CHTx	Alive, under surveillance
2	16	M	Weight loss, bloody stool	12	Rectal solid mass	R	None	MA	Refused Tx	Lost to follow-up
3	15	M	Weight loss, bloody, mucoid stool	6	Rectal solid mass	R	None	MA	STx+CHTx	Exitus
4	16	F	Bloody, mucoid stool, anemia	2	Solid mass in descending colon	RS, DC	None	MA + TA	STx+CHTx+ RTx	Remission
5	15	M	Weight loss, Bloody stool	4	Solid mass in descending colon	DC	None	MA	STx+CHTx	Remission

M indicates male; F, female; R, rectum; RS, rectosigmoid; DC, descending colon; PDA, poorly differentiated adenocarcinoma; MA, mucinous adenocarcinoma; TA, tubular adenocarcinoma; STx, surgical therapy; CHTx, chemotherapy; RTx, radiotherapy.

## DISCUSSION

CRC in childhood is very rare, usually diagnosed at an advanced stage and often has poor prognosis (5). CRC in children are mostly sporadic, roughly 10% of cases may have a predisposing condition (6). The most common predisposing factors are familial adenomatous polyposis, hereditary nonpolyposis colorectal cancer, Gardner syndrome, Turcot syndrome, Peutz-Jeghers syndrome, juvenile polyposis of colon, and ulcerative colitis (7). There was no family history of consanguinity or colonic cancer in our patients, but a mutation in the DNA mismatch repair gene was detected in case 4 with a family history of adenomatous polyposis. It has been suggested that genetic analysis for the inherited mutations should be considered in cases of early-onset CRC (7), and the absence of genetic analysis in 4 patients may be a drawback of this study.

Abdominal pain is one of the most reported presenting symptoms in pediatric CRC patients (3,4). Abdominal distention, altered bowel habits, and rectal bleeding were also reported less frequently as well as weight loss (3). At presentation, bloody stool was a common symptom in all of our cases. Since rectal bleeding is a distressing and indisputable symptom, abdominal pain might have been ignored. Another explanation was that the mass within the colonic lumen might not be large enough to cause distention and induce abdominal pain in our patients. Pediatric CRC patients are usually diagnosed at an advanced stage and more than half of the children with CRC would have metastases at presentation (6). Nonspecific complaints such as abdominal pain or constipation before the emergence of remarkable symptoms usually impede the early diagnosis, and the disease may evolve into advanced stages with a poor prognosis.

Recent guidelines on colorectal cancer screening recommend fecal occult blood or fecal immunochemical DNA examinations in adults (8). Lately, there have been several studies investigating the diagnostic accuracy of fecal calprotectin (FC) for CRC screening. A recent meta-analysis concluded that FC cannot be recommended for CRC detection yet (9). Since all of the patients presented with rectal bleeding in this study, colonoscopy was the first diagnostic method performed, and FC was not analyzed in our patients. Computed tomography (CT) or MRI are usually used either to evaluate the abdominal symptoms at presentation or to detect metastasis in CRC patients (10). We performed an abdominal MRI in all patients, and liver metastasis was discovered in one patient.

Treatment strategies of CRC in children are usually derived from adult data (10). Cure and prognosis usually depend on total excision of the tumor; however, this is not the case in majority of childhood CRC patients. Patients with limited disease may be treated with surgery alone. Neoadjuvant chemotherapy can be tried to reduce the tumor bulk or metastatic lesions before the surgical resection. Adjuvant multi-agent chemotherapy is necessary for both early and advanced stage CRC. Patients with solitary metastatic lesion, surgical resection of both tumor and metastases is recommended. Radiotherapy is reserved for rectal cancer in combination to surgery and chemotherapy or as a palliative treatment (10). Case 1 underwent a surgical resection, and was given postoperative adjuvant chemo and radiotherapy, but liver metastasis did not respond to the treatment. He is still alive and being followed by an oncologist. Two patients were operated after receiving neo-adjuvant chemotherapy, and one of them was alive. No relapse was observed during the follow-up of our CRC patients.

In conclusion, although it is rare, childhood CRC should be considered in any children with unexplained abdominal pain, weight loss, persistent constipation, abdominal distension/obstruction, or rectal bleeding. Not only the aggressive nature of childhood CRC but also the lack of awareness of early onset of the disease may defer diagnosis and facilitates distant metastasis.
